# Patient reported quality of life in young adults with sarcoma receiving care at a sarcoma center

**DOI:** 10.3389/fpsyg.2022.871254

**Published:** 2022-09-29

**Authors:** Jonathan R. Day, Benjamin Miller, Bradley T. Loeffler, Sarah L. Mott, Munir Tanas, Melissa Curry, Jonathan Davick, Mohammed Milhem, Varun Monga

**Affiliations:** ^1^Department of Internal Medicine, Carver College of Medicine, University of Iowa, Iowa City, IA, United States; ^2^Department of Orthopedics, Carver College of Medicine, University of Iowa, Iowa City, IA, United States; ^3^Holden Comprehensive Cancer Center, Iowa City, IA, United States; ^4^Department of Pathology, Carver College of Medicine, University of Iowa, Iowa City, IA, United States

**Keywords:** quality of life, sarcoma, young adult, oncology-discipline, FACT-G

## Abstract

**Background:**

Sarcomas are a diverse group of neoplasms that vary greatly in clinical presentation and responsiveness to treatment. Given the differences in the sites of involvement, rarity, and treatment modality, a multidisciplinary approach is required. Previous literature suggests patients with sarcoma suffer from poorer quality of life (QoL) especially physical and functional wellbeing. Adolescent and young adult (AYA) patients are an underrepresented population in cancer research and have differing factors influencing QoL.

**Methods:**

Retrospective analysis of Young Adult patients (age 18–39) enrolled in the Sarcoma Tissue Repository at University of Iowa. QoL was assessed using the self-report FACT-G questionnaire at enrollment and 12 months post-diagnosis; overall scores and the 4 wellbeing subscales (Physical, Emotional, Social, Functional) were calculated. Linear mixed effects models were used to measure the association between the rate of change in FACT-G subscale scores and baseline clinical, comorbidity, and treatment characteristics.

**Results:**

49 patients were identified. 57.1% of patients had a malignancy involving an extremity. Mean FACT-G scores of overall wellbeing improved from baseline to 12 months (76.4 vs. 85.4, *p* < 0.01). Social and emotional wellbeing did not differ significantly between baseline and 12 months. Physical wellbeing (18.8 vs. 23.9, *p* < 0.01) and functional wellbeing (16.8 vs. 20.0, *p*< 0.01) scores improved from baseline to 12 months. No difference was seen for FACT-G overall scores for age, sex, laterality, marital status, performance status, having children, clinical stage, limb surgery, chemotherapy, or tumor size. A difference was demonstrated in physical wellbeing scores for patients with baseline limitation (ECOG 1-3) compared to those with no baseline limitation (ECOG 0) (*p* = 0.03). A difference was demonstrated in social wellbeing based on anatomical site (*p* = 0.02).

**Conclusion:**

Young adults with sarcoma treated at a tertiary center had improvements in overall reported QoL at 12 months from diagnosis. Overall baseline QoL scores on FACT-G were lower than the general adult population for YA patients with sarcoma but at 12 months became in line with general population norms. The improvements seen merit further investigation to evaluate how these change over the continuum of care. Quality of life changes may be useful outcomes of interest in sarcoma trials.

## Introduction

Though underrepresented in research approximately 89,500 adolescents and young adults (AYA) are diagnosed with cancers in the United States (US) annually (Miller et al., 2020). Sarcomas are among the most common cancers in the AYA age group, age 15–39 as defined by the National Cancer Institute with an incidence rate between 1.3 and 3.6 per 100,000 for soft tissue sarcomas and 0.3–1.6 per 100,000 for bone cancers (Miller et al., 2020). Sarcomas are a diverse group of neoplasms that vary greatly in clinical presentation and responsiveness to therapy (Hui, 2016). Given this medical and scientific complexity, and a heterogenous population in terms of sites of involvement rarity, age groups effected, and treatment modalities, a multidisciplinary approach is required with a focus on patient centered care and patient quality of life (Bottomley, 2002; Soliman et al., 2009; Deshpande et al., 2011; Winnette et al., 2017).

Previous literature suggests patients with sarcoma suffer from poorer quality of life (QoL) especially regarding physical and functional wellbeing (Coens et al., 2015; Hudgens et al., 2017). Similarly poor quality of life outcomes in terms of physical and functional wellbeing were seen for AYA patients with sarcoma in AYA HOPE study (Smith et al., 2019). Age groups are affected differently both in terms of the type of sarcoma they have and how this impacts their lives (van der Graaf et al., 2017). QoL in AYA patients may have more of an impact on studies, jobs, and changes in social relationship during the course of their treatment (Fujii et al., 2019). They may also recognize differences in QoL more reliably than their providers (Kaal et al., 2021).

Recent published reviews acknowledge the paucity of literature on quality of life and psychosocial issues in patients with sarcoma (McDonough et al., 2019). There is even less literature regarding AYA patients with sarcoma. Therefore, this study aims to understand if there is an association with treatment at a tertiary sarcoma center and differences in quality of life for young adults with sarcomas.

## Materials and methods

### Study design and population

A retrospective analysis was undertaken of young adult (YA) patients with sarcoma age 18–39 in the Sarcoma Tissue Repository (STiR) who had an available enrollment questionnaire or 12-month questionnaire wherein at least one of the subscales was completed. Patients under the age of 18 were not enrolled in the registry as patients under 18 are only asked about enrollment at physician request therefore the standard definition of AYA per NCI was not used (Adolescent and Young Adult Oncology Progress Review Group [AYAOPR]., 2006). Data was obtained from review of the University of Iowa Oncology Registry and Sarcoma Tissue Repository (STiR) established in 1992, as well as electronic medical records. Patients were selected who were enrolled in the Sarcoma Tissue Registry and received care at University of Iowa between 2008 (when FACT-G administration began) and 2021. Only patients who had a baseline/enrollment FACT-G filled out within 6 months of diagnosis were included in the study. The planned focus of the study was short term follow up to next 1 year questionnaire looking at patient, disease, treatment, and QoL data.

### Demographics

Eastern Cooperative Oncology Group (ECOG) performance status scores were retrieved from clinical notes if reported and if not reported were assigned by a clinician based on information provided in the chart at baseline. Information present in history, exam, or questions regarding activity were used by clinicians to help assess performance. Clinical stage was abstracted from the patient chart and was assigned using NCCN guidelines for the specified site by clinician review if not initially reported.

### Outcomes

QoL was assessed using self-reported Functional Assessment for Cancer Therapy-General (FACT-G) questionnaires at baseline enrollment and 12 months. The FACT-G is a well validated, 27 question, 104 point scale that has four subscales assessing physical wellbeing (PWB, 0–28), functional wellbeing (FWB, 0–28), social/family wellbeing (SWB, 0–28), and emotional wellbeing (EWB, 0–24) (Cella et al., 1993; Victorson et al., 2008). Scores were reported as overall wellbeing and 4 subscales; physical, emotional, social and functional.

### Analysis

Linear mixed effects models were used to estimate the overall change in QoL scores between enrollment and 12-month and measure the association between the rate of change in FACT-G QoL scores and patient (e.g., age, gender, marital status), disease (e.g., stage, grade), and treatment (e.g., biopsy, surgery, chemotherapy, radiation) characteristics. Random effects were included to account for the longitudinally correlated nature of repeated QoL assessments at unequal time spacing between visits with a spatial power correlation structure. All statistical testing was two-sided and assessed for significance at the 5% level using SAS v9.4 (SAS Institute, Cary, NC).

This study was reviewed and approved by the University of Iowa Intuitional Review Board (IRB 202106171).

## Results

### Demographics

A total of 49 young adult (YA) patients met inclusion criteria for the study. There were 21 (42.9%) female patients. Age at diagnosis had a mean of 29 years [standard deviation (SD) = 6.4 years] and ranged from 19 to 39 years. The patients were predominantly Caucasian (98.0%). Nearly one half of patients had no limitations due to malignancy at baseline, ECOG = 0 (49.0%). Married patients made up 42.6% of the population. Of young adults with sarcoma 28.6% had children (Table 1). Though the study allowed for patient data as far back as 1992, the earliest patient date of diagnosis was 2008.

**TABLE 1:** Demographics.

Variable	Level	*N* = 49
Sex	Female	21 (42.9)
	Male	28 (57.1)
Race/Ethnicity	Caucasian	48 (98.0)
	Hispanic	1 (2.0)
Laterality	Left	17 (34.7)
	Not paired	16 (32.7)
	Right	16 (32.7)
Performance status (enrollment)	0	24 (49.0)
	1	18 (36.7)
	2	6 (12.2)
	3	1 (2.0)
Performance status	0	24 (49.0)
	1–3	25 (51.0)
Marital status	Married	20 (42.6)
	Single	27 (57.4)
	Missing	2
Has biological children	No	35 (71.4)
	Yes	14 (28.6)
Clinical stage	1	14 (31.8)
	2	13 (29.5)
	3	6 (13.6)
	4	11 (25.0)
	Missing	5
Clinical stage	1–2	27 (61.4)
	3–4	17 (38.6)
	Missing	5
Location	Abdomen	6 (12.2)
	Head	4 (8.2)
	Lower Ext	22 (44.9)
	Other	1 (2.0)
	Pelvis	6 (12.2)
	Thoracic	4 (8.2)
	Upper extremity	6 (12.2)
Trunk vs. Extremity	Extremity	28 (63.6)
	Trunk	16 (36.4)
	Missing	5
Surgery w/in first year	No	3 (6.1)
	Yes	46 (93.9)
Limb surgery w/in first year	No	23 (46.9)
	Yes	26 (53.1)
Type of limb surgery	Amputation	2 (7.7)
	Limb-Sparing	24 (92.3)
	Missing	23
Radiation w/in first year	No	40 (81.6)
	Yes	9 (18.4)
Chemotherapy/targeted/hormone therapy w/in first year	No	17 (34.7)
	Yes	32 (65.3)

Distribution by stage of cancer varied widely with 31.8% having stage I, 29.5% having stage II, 13.6% with stage III, and 25.0% with Stage IV disease per NCCN guidelines (Network Ncc. Nccn, 2021a,b,c). More YA patients had sarcoma of the extremity (57.1%%) than of the trunk (32.6%). The most frequently reported location of disease was the lower extremity 44.9% (Table 1).

### Treatment

93.9% of patients underwent surgery within 1 year of diagnosis. Over one half (53.1%) of patients had limb surgery. Of those who had limb surgery 92.3% had limb-sparing surgery over amputation. 34.7% of patients underwent chemotherapy, hormone therapy, or targeted therapy and only 18.4% had radiation within the first year of diagnosis. All patients who underwent radiation in the first year received it as an adjuvant therapy (Table 1).

### Patient reported quality of life by FACT-G

Self-reported FACT-G scores were recorded for 37 patients at baseline and 27 patients at 12 months (Table 2). The median time from diagnosis to completion of baseline questionnaire was 1 month and 12 months for the 1 year follow up survey. Differences between total eligible patients and self-reported FACT-G’s existed due to various circumstances. Only 1 patient was lost to follow-up during this time period, resulting in 1 missing 12-month questionnaire. In addition, 1 baseline questionnaire was completed outside of the defined time window (within 6-months of the intended completion date). The remaining questionnaires at the respective time points are missing because patients did not return a completed questionnaire for unknown reasons.

**TABLE 2:** FACT-G wellbeing scores.

	Questionnaire		
		
Covariate	Enrollment	Change (12-month–Enrollment)	*P-value
Physical wellbeing	18.8 (16.6, 21.0)	5.1 (2.2, 8.0)	**<0.01**
Social wellbeing	24.4 (23.0, 25.8)	-0.6 (-2.5, 1.4)	0.56
Emotional wellbeing	17.1 (15.6, 18.6)	0.3 (-1.2, 1.8)	0.66
Functional wellbeing	16.5 (14.6, 18.3)	3.3 (1.1, 5.5)	**<0.01**
Overall wellbeing	76.4 (71.3, 81.4)	9.0 (3.5, 14.5)	**<0.01**

*P-values are from linear mixed effects models. ^†^22 patients had only a baseline questionnaire. 15 patients had both a baseline and 12-month questionnaire. 12 patients had only a 12-month questionnaire. Bold values are statistically significant

**FIGURE 1:**
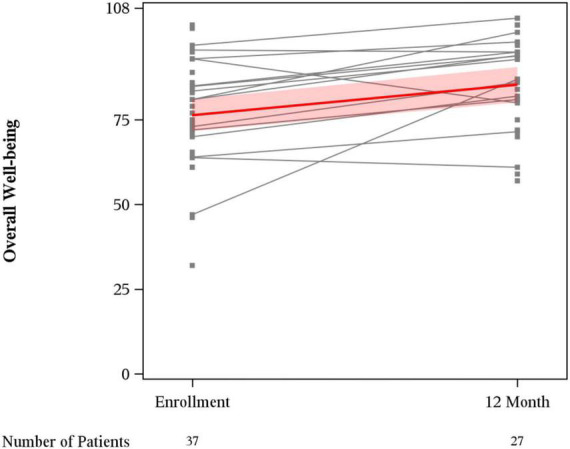
FACT-G overall wellbeing score.

**FIGURE 2:**
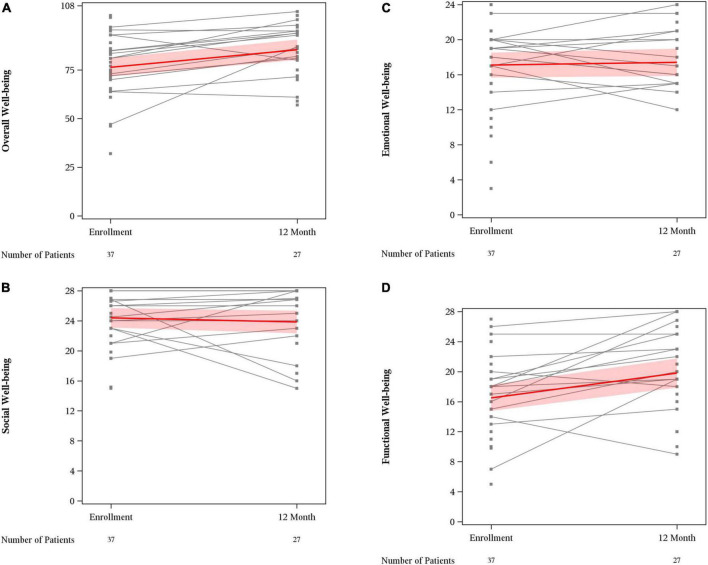
FACT G subset scores. **(A)** Overall well-being; **(B)** social well-being; **(C)** emotional well-being; and **(D)** functional well-being.

Mean overall wellbeing scores also improved from baseline to 12 months (76.4 vs. 85.4, *p* < 0.01) (Figure 1). Physical wellbeing differed significantly between baseline reports and 12 months (18.8 vs. 23.9, *p* < 0.01) (Figure 2). Social wellbeing and emotional wellbeing did not differ significantly between baseline and 12 months; *p* = 0.56 and *p* = 0.66 respectively. Mean functional wellbeing scores were 16.5 at baseline and 19.8 at 12 months showing significant increase (*p* < 0.01) (Figure 2).

**FIGURE 3:**
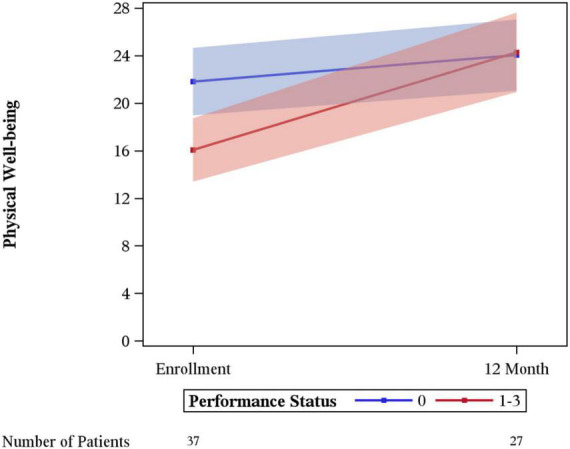
Changes in physical wellbeing by performance status.

**FIGURE 4:**
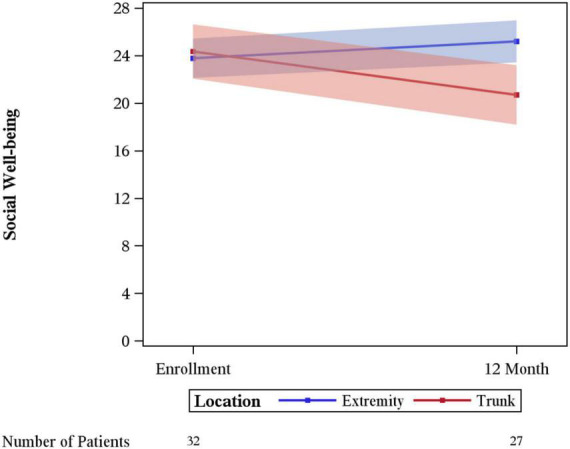
Changes in social wellbeing by anatomic location.

Significant differences in the rate of change between enrollment and 12-month FACT-G overall scores by age, sex, laterality, marital status, performance status, having children, clinical stage, limb surgery, chemotherapy, or tumor size were not evidenced. A statistically significant difference was seen for physical wellbeing among patients with no limitations (performance status of 0) vs. some limitation (performance status of 1–3) (*p* = 0.03), with those with some limitation showing a greater degree of improvement (Figure 3). A difference in social wellbeing scores was also seen based on location of malignancy, trunk vs. extremity (*p* = 0.02), with those with extremity showing greater improvement in scores (Figure 4).

## Discussion

This retrospective review from an academic tertiary care center reveals that young adults with sarcoma report a perception of improved quality of life at 12 months after their diagnosis. Patients with decreased functional status at baseline report a higher change in quality of life, approaching those who did not have baseline limitations. Importantly, a difference is seen in changes in reported perception of social wellbeing depending on anatomical location with patients, with sarcomas of the trunk reporting decreased social wellbeing and patients with extremity sarcomas reporting improvements in social wellbeing. To our knowledge this analysis represents the largest study reviewing QoL outcomes in YA patients with sarcoma.

Overall FACT-G scores at enrollment were in line with non-GIST sarcoma scores reported in adults with a mean of 76.4 in this study and previous literature for adults with sarcoma reporting 76.4 and 75.49 (Ostacoli et al., 2014; Chan et al., 2015). Physical, Emotional, and Functional FACT-G scores were in line with previously reported study (Ostacoli et al., 2014). Social wellbeing in this YA cohort 24.4 were numerically higher than in cohorts of all adults 19.04 (Ostacoli et al., 2014).

**FIGURE 5:**
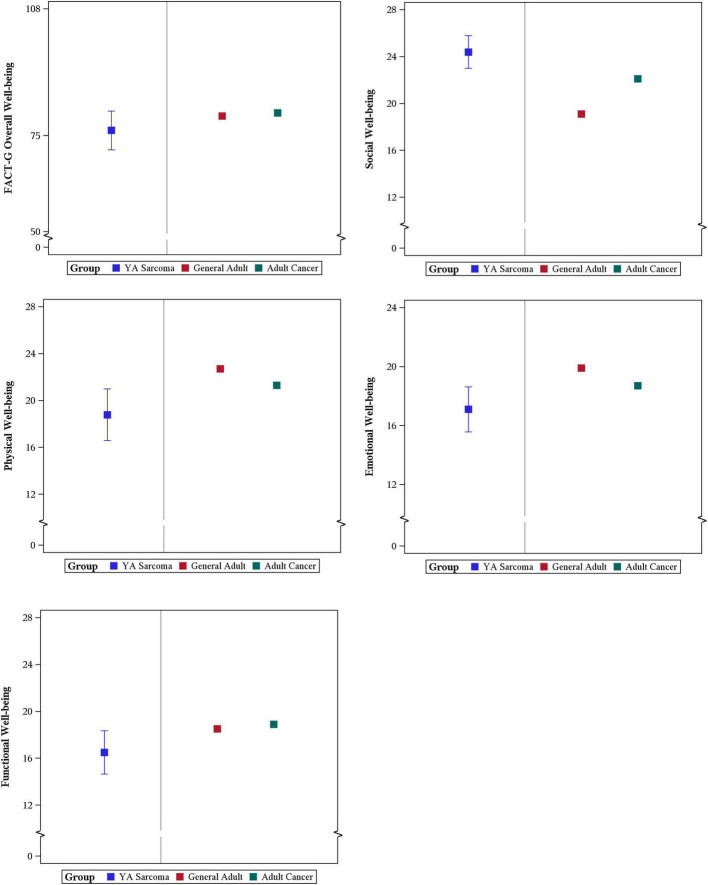
Comparison of YA patient reported FACT-G scores with General Adult and Adult with Cancer Population Scores.

When compared with childhood cancer survivors, physical wellbeing was numerically lower but with greater variability in the YA sarcoma patients in this study with a mean physical wellbeing of 18.8 in survivors of childhood cancer. In YA patients with sarcoma, baseline physical wellbeing scores are much lower than the general population with a mean of 25.1. social wellbeing scores seen in this YA sarcoma cohort are in line with AYA survivors, and higher than the United States general population mean 19.1 (Brucker et al., 2005; Bradford et al., 2021; Figure 5).

Physical wellbeing showed improvement in patients between their baseline and 1 year follow up. This could potentially be explained with treatment effect, and relief of pain, and limitations due to the location of their primary malignancy. Improvements in physical wellbeing scores were noted mostly in those who had decreased ECOG performance status at baseline. One could surmise that patients may have regained function and with treatment of their sarcoma noticed this improvement. In this study few patients underwent amputation and most underwent limb sparing procedures at a high-volume center which has been shown to be associated with improved overall survival (Abarca et al., 2018).

Changes in social wellbeing had significant variation between patients with extremity vs. trunk sarcoma. This may relate to the improvements in wellbeing from being more active and able to be socially engaged after treatment of an extremity sarcoma. Limb sparing surgery which was done in >90% of the patients in this study may make this easier for patients with extremity sarcoma. Whereas patients with trunk sarcomas may require undergoing large surgical revisions and have significant scarring. This could be a source of embarrassment in some social situations. Other factors may depend on the extent of involvement of the sarcoma for example if there was bowel resection, or other organs were removed. Patients may have changes in intimate relationships or apprehension regarding sexual activity and body imaging post-surgical and chemotherapy treatments. Additionally, it is interesting that YA patients with sarcoma have numerically higher scores than age-matched peers or even age-matched peers with other malignancies (Brucker et al., 2005). This may be reflective of patients reaching out for support surrounding their diagnosis or is potentially reflective of the nature of the local culture.

For treating providers, clinical outcomes, such as overall survival and progression, seem paramount. One must not lose sight of other outcomes that matter to patients such as quality of life. Differences in patient-reported outcomes such as quality of life become important to track over time and should help serve as important endpoints for clinical trials in conjunction with subjective measures (Osoba, 2011). Ideally this will include longer follow up into patient survivorship to gain insight on the patient experience not just around initial treatment but how patients are cared for in the long term (Haslam et al., 2020).

This study has several important limitations that must be acknowledged. First this study is limited to the YA age group 18–39 and did not include adolescents; as patients ages 15–17 are not included in STiR at the University of Iowa. This study represents the experience of a single tertiary academic center and had a limited population that lacked diversity and may not be applicable to all patients. A limited sample size makes it difficult to understand the role of various factors in QoL. However, this limited data serves to aid in hypothesis generation for this understudied group. A response bias must also be acknowledged as some patients who are doing worse or are very ill may not have sent back surveys, possibly underestimating the true QoL in this population. Given that there is some variability for baseline enrollment and survey response there may be a concern of recall bias, this should be limited in that QoL is the only self-reported measure and all other data was derived from medical records. The patients that responded to the surveys may not be reflective of all patients.

## Conclusion

Young adults with sarcoma treated at a tertiary center had improvements in overall reported QoL at 12 months. Overall baseline QoL scores on FACT-G were lower than the general population for YA patients with sarcoma but at 12 months were in line with general population norms. The improvements seen merit further investigation to evaluate how these change over the continuum of care and if interventions are needed at specific timepoints. Quality of life changes may be useful outcomes of interest in trials.

## Data availability statement

The raw data supporting the conclusions of this article will be made available by the authors, without undue reservation.

## Ethics statement

This study was reviewed and approved by the University of Iowa Intuitional Review Board (IRB 202106171). Written informed consent for participation was not required for this study in accordance with the national legislation and the institutional requirements.

## Author contributions

JRD, BL, SM, BM, and VM contributed to conception and design of the study. JRD, MC, BL, and SM organized the database. BL and SM performed the statistical analysis. JRD wrote the first draft of the manuscript. JRD, VM, BL, SM, and BM wrote sections of the manuscript. All authors contributed to manuscript revision, read, and approved the submitted version.
